# Performance Effects with Injury Prevention Exercise Programmes in Male Youth Football Players: A Randomised Trial Comparing Two Interventions

**DOI:** 10.1186/s40798-020-00282-7

**Published:** 2020-11-23

**Authors:** Hanna Lindblom, Markus Waldén, Martin Hägglund

**Affiliations:** 1 Unit of Physiotherapy, Division of Prevention, Rehabilitation and Community Medicine, Department of Health, Medicine and Caring Sciences, Linköping University, Linköping, Sweden; 2 Sport Without Injury ProgrammE (SWIPE), Department of Health, Medicine and Caring Sciences, Linköping University, Linköping, Sweden; 3 Unit of Public Health, Division of Society and Health, Department of Health, Medicine and Caring Sciences, Linköping University, Linköping, Sweden; 4 Department of Orthopaedics, Hässleholm-Kristianstad Hospitals, Hässleholm, Sweden

**Keywords:** Soccer, Neuromuscular training, Adolescents

## Abstract

**Background:**

Increased performance from injury prevention exercise programmes (IPEPs) may affect injury risks positively and support the implementation of IPEPs. The primary aim was to study the performance effects of injury prevention exercises from two different IPEPs, the *Knee Control* IPEP and the further developed *Knee Control+* IPEP, in youth male football players, and the secondary aim was to compare potential differences in performance effects between the IPEPs.

**Methods:**

Four male youth football teams were tested for agility, hop and sprint performance at the start of the second half of the competitive season and after the end of the 8-week season. Per randomisation, two teams used *Knee Control* and two teams *Knee Control+*.

**Results:**

In total, 47 players executed a median of 13 IPEP sessions (range 11–21 sessions). No improvements in performance were seen in the group as a whole. The intervention groups showed small declines in sprint and agility performance. There was a significant between-group difference in change for the 505 agility test, with improved performance in the *Knee Control* and worse performance in the *Knee Control+* group, Δ_KC vs KC+_ = − 0.012 (95% CI − 0.19 to −0.04), *d* = 0.98.

**Conclusions:**

No clinically meaningful performance effects were seen from the *Knee Control* or *Knee Control+* IPEP in youth male athletes and no meaningful differences were seen between *Knee Control* and *Knee Control+* regarding effects on performance tests.

**Trial registration:**

ClinicalTrials.gov identifier: NCT03251404. Registered on 16 August 2017.

## Key Points

- No meaningful effects on performance were seen from the *Knee Control* or *Knee Control+* programmes after 8 weeks of training
- No meaningful differences were seen in effects on performance between programmes
- Even though the *Knee Control* and *Knee Control+* interventions were introduced during a period of heavy overall training load, performance was not reduced, as assessed in agility, hop and sprinting tests among male youth football players after 8 weeks of training

## Introduction

High performance in competitive sports is important for obvious reasons. There are two primary reasons for studying performance effects of injury prevention exercise programmes (IPEP): (a) improved performance may affect injury risks positively [1] and (b) improved performance may be a key component to improve programme adherence and succeeding with real-life implementation [1]. Boosting adherence and utilisation fidelity is an important goal in itself since previous studies on different IPEPs have had difficulties with attaining high adherence with the programme protocol [2–5], potentially limiting the preventive effect. Additionally, learning more about the effects on performance will likely increase our understanding also of the effect mechanisms behind the injury risk reduction of the preventive programmes [6], which are not fully understood at present [7, 8].

A recent meta-analysis including 14 studies on different IPEPs, predominantly in football players, showed positive performance effects favouring the interventions regarding balance/postural stability, strength, sprint ability and speed [6]. The effects were only small to moderate in general but with large positive effects for leg strength and sprint abilities in male youths [6]. Studies of the 11+ and 11+ Kids, IPEPs known to reduce injuries in football, have shown positive effects in males on various tests of agility [1, 9, 10], vertical jump height [1, 9, 10], balance/stability [11] and strength [12–14]. When comparing results across studies, the results are, however, inconclusive with studies showing effects versus lack of effects on different tests and showing positive effects also in the control group indicating that all changes are not related to the intervention per se [9–11, 14].

*Knee Control* (SISU Idrottsböcker©, Sverige, 2005) is a coach-led IPEP developed for team ball sports, originally available on a CD-ROM but from 2012 with added running warm-up on a mobile application/webpage. *Knee Control* was highly efficacious in reducing the risk of anterior cruciate ligament injuries in female youth football players [15]. However, many coaches report that they modify the IPEP to improve programme fit and increase player buy-in [3, 16], but with risk of compromising the preventive effect. Based on this knowledge, *Knee Control+* was developed to improve programme fidelity. Eight physiotherapists and one medical doctor, all with several years of experience from using the *Knee Control* IPEP and educating players and coaches in the *Knee Control* IPEP, took part in this work.

Despite all attention that prevention has received among females, the adherence and fidelity have been low [3, 16] and focusing on performance effects instead may provide additional incentive to participate in preventive training. This is probably of even greater importance in male teams since injury prevention has not received as much attention among youth male players. The performance effects of the original *Knee Control* IPEP have previously been studied in youth female football players, without any apparent positive effects after 11 weeks of training [17]. In that study, the IPEP training dose was low because of low player attendance at team training sessions and thus resulting in an overall poor IPEP compliance. Performance effects of *Knee Control* have not been studied in youth male players previously, and performance effects of *Knee Control+* have not been studied at all.

### Objective

The primary aim was to study the performance effects of injury prevention exercises from two different IPEPs, *Knee Control* and *Knee Control+*, in youth male football players, and the secondary aim was to compare potential differences in performance effects between the IPEPs.

Our hypothesis was that there would be a performance effect from using both IPEPs. We expected similar effects of both programmes, or superior effects of *Knee Control+* if the adherence was higher in this group.

## Material and Methods

This was a randomised trial where we used stratified block-randomisation of teams to each intervention arm to avoid contamination between groups. The study was also part of a pilot feasibility study evaluating *Knee Control+* for the first time. The study was carried out in Linköping, Sweden, during the second half of the competitive season between the middle of August after schools started and end of October 2017 when the outdoor season ended. Data collection was done at baseline and at follow-up after approximately 8 weeks of training. The study adheres with the CONSORT guidelines. The study was registered on ClinicalTrials.gov before study commencement (trial registration number: NCT03251404).

### Participants

A convenience sample of four male youth football teams was included. Eligible teams had players aged 13–17 years and scheduled football training at least twice per week. In total, 81 players aged 13–16 years were eligible for inclusion in the four teams. For inclusion, the players had to be physically healthy, injury-free and able to participate in performance testing with maximum effort. Background information about the players, such as current training volume and participation in other sports, was collected through questionnaires at baseline. The study was approved by the regional ethical review board in Linköping, Sweden: Dnr 2017/294-31. Players and their legal guardians received written information about the study and signed written informed consent forms before study commencement. They were informed that all data would be fully anonymised and that they cannot be identified via the paper. The study was performed in accordance with the Declaration of Helsinki.

### Intervention

Two different versions of an IPEP were used: (a) the *Knee Control* IPEP mobile application/webpage version and (b) the *Knee Control+* IPEP (Appendix). Both programmes contain a standardised running warm-up (5 min) and the same six principal exercise components (approximately 15 min): one-legged knee squats, hamstring strengthening, two-legged knee squats, core strength, lunges and jump/landing. *Knee Control+* contains more progressions for the coach to be able to adapt the programme content to fit their team (e.g. for younger or older junior players) and for greater exercise variation with 30 more progressions for the 6 principal exercises in addition to the 30 progressions that already exist in the original programme. Additionally, due to the more advanced exercise options with added alternatives for hamstring strengthening such as the Nordic hamstrings and plyometric alternatives to two-legged knee squats and lunges, the potential for positive effects may be greater.

All team coaches received written and oral information about one of the IPEPs (as per randomisation) and practical instructions from physiotherapists during a team training session. When possible both coaches and players received practical education at the same time, otherwise, the coaches were given practical education and then introduced the intervention to the players themselves. The teams were instructed to use the allocated programme during the warm-up at every training session during the study period. All coaches were recommended to start with the easiest versions of the six exercises and were allowed to progress as early as two weeks after training commenced with the approval of the first author. If a coach reported that players had difficulties reaching the goal of an exercise, the first author suggested a similar alternative exercise from the same programme. The coaches were instructed to record each training session if the IPEP had been used and individual player participation, and any adverse events.

### Testing Procedures

One team at a time was tested at baseline and follow-up. The aim was to test all teams at the same time of day at baseline and follow-up, which was possible in three of the teams, whereas the last team was tested after lunch at baseline and in the evening at follow-up. Testing was done indoors in the same venue, and players were asked to refrain from physically exhausting training on the day before testing. Prior to testing, all players took part in a standardised running warm-up for 5 min, the same as used prior to the IPEPs, led by two physiotherapy students.

The test battery included tests of agility, hop and sprint performance as well as jump-landing technique used in the following order: drop vertical jump, agility t test, single-leg hop for distance, 505 agility test, side-hop test, 10-m and 20-m sprint test, tuck jump assessment and countermovement jump test (CMJ). Six of the tests were used primarily to evaluate sport-specific performance and are reported in the present paper (Table 1). The drop vertical jump and tuck jump assessment were used to evaluate jump-landing technique and are reported elsewhere [23]. The tests were chosen to represent different aspects of performance and to represent different modes of agility (turning, side-shuffling, acceleration and deceleration, etc.) and jump ability (vertical jump, horizontal jump, repetitive jumps). Additionally, these tests were chosen because we believed they were feasible to carry out in this youth population. The testing order of the players was the same during all tests, and it took about 2 h to complete the test battery for the whole team. Players were allowed as much recovery between tests as needed to be able to perform with maximum effort, a range of rest breaks of 30 s to 2 min. All players were recommended to wear tight shorts, t-shirt, short socks and indoor shoes. Five players in one of the teams chose to perform the tests barefoot at both baseline and follow-up. Two physiotherapists and two physiotherapy students were responsible for the testing on all testing occasions, and all six performance tests were led by assessors blinded to group allocation. The best test result was used in the analyses.

**Table 1:** Description of the included performance tests

Performance test	Test description	Disqualified test	Equipment	No of practice trials	No of test trials^**b**^
Agility t test: For change of direction agility	The player ran 10 m forwards towards a cone, side shuffled 5 m to the left, touched a cone with the left hand, side shuffled 10 m to the right and touched a cone with the right hand and then side shuffled 5 m to the left and touched the middle cone with the left hand before sprinting 10 m backwards to the starting position. Testing started on command. The test had excellent test-retest reliability, ICC 0.98, in youth male football players [18].	The test was repeated if the player crossed his legs during side shuffling.	Timing gates^a^	≥ 2	2
Single-leg hop for distance: For maximal horizontal hop performance	The player started standing on one leg and hopped as far as possible and landed on the same leg. Free leg-swing was allowed and a balanced landing, where the player could stand still for 2–3 s, was sought. Hands were kept on the back during the entire test. The test had good test–retest reliability, ICC 0.80, in male and female recreational athletes [19]	The test was repeated if a player lost balance or failed to remain standing on one leg upon landing.	Tape measure	≥3 per leg	3 per leg
505 agility test: For acceleration and speed during a 180° turn	The test was performed as described by Draper and Lancaster [20]: the player sprinted 15 m forwards, through timing gates positioned after 10 m, made a 180° turn at the 15-m line and sprinted 5 m back through the same timing gates again. The test had excellent test-retest reliability, ICC 0.95, in female netball players [21].	The test was repeated if none of the feet crossed the 15-m line.	Timing gates^a^	≥ 2	2
Side-hop test: For hop endurance	The player stood on one leg with the hands on the back and hopped as many times as possible for 30 s between two tape markings 40 cm apart. The number of approved hops was counted afterwards using films. The test had good test–retest reliability, ICC 0.72, in healthy males [22].	Hops were not counted if the player’s foot touched the marking or landed between the markings, or if he lost balance and put the other foot on the floor.	Tests were filmed using two GoPro Hero5 cameras	Free practice	1 per leg
10- and 20-m sprint: For sprint performance	The player ran through timing gates positioned at the start, at 10 m and at 20 m. Testing started on command. The test had excellent test–retest reliability, ICC 0.96 for 10 m sprints and ICC 0.95 for 20-m sprints, in youth male football and handball players [18].	The test was repeated when needed.	Timing gates^a^	1	2
Countermovement jump: For vertical jump performance	The test was performed with the hands on the hips and without an overhead target. The player started the test by making a squat immediately followed by a vertical jump and landing with straight legs. The test had excellent test–retest reliability, ICC 0.94, in youth male football and handball players [18].	The test was repeated if the player did not jump and land as described.	Infrared contact mat^a^	≥2	3

*ICC* intraclass correlation coefficient

^a^MuscleLab 4010, Ergotest Technology a.s., Norway

^b^The best test result was used in the analyses

### Statistical Analysis

A sample size calculation based on the mean and standard deviation of the agility t test [19] showed that to achieve a power of 80% at an alpha-level of 0.05 and an estimated 5% improvement between baseline and follow-up, a total of 28 players were needed. With an estimated drop-out rate of 20% between baseline and follow-up, we aimed to recruit at least 34 players. SPSS Statistics for Windows (version 24.0, IBM) was used for the analyses. The primary outcome measure was changed in the agility t test.

The significance level was set at *p* < 0.05. Mixed design ANOVAs were used to analyse the effect over time in (1) the whole sample, irrespective of group allocation, and (2) for within-group and between-group comparisons between *Knee Control* and *Knee Control+* on each performance test, adjusted for age and using all complete cases with both baseline and follow-up data. For the between-group comparisons effect sizes, partial eta-squared, were calculated and converted to Cohen´s *d*. Effect sizes were interpreted as small *d* = 0.2, medium *d* = 0.5 and large *d* = 0.8 [24]. A sensitivity analysis including all players assessed at baseline was made, using a mixed model, and compared to the complete case analysis.

## Results

**Fig. 1:**
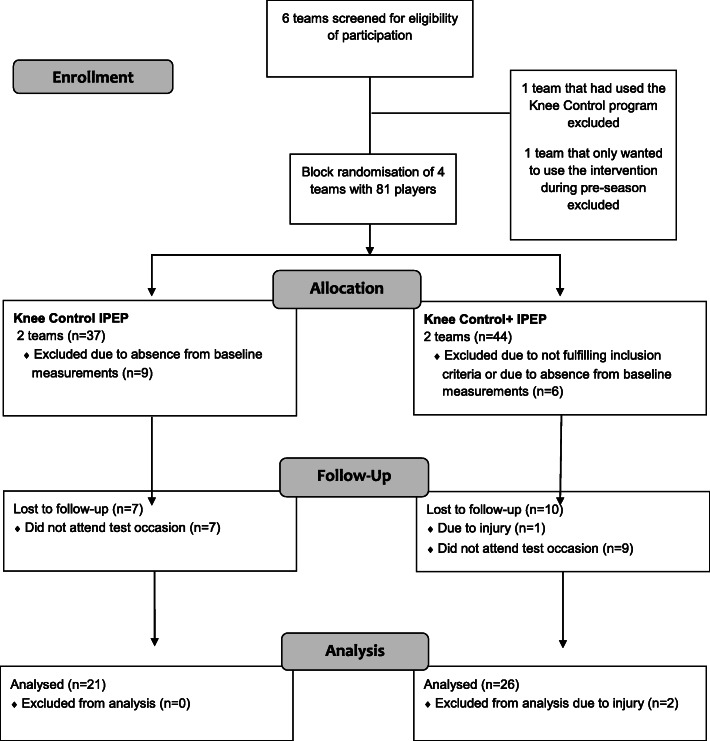
Consort flow chart describing the inclusion of players in the different phases of the trial

At baseline, 66 players took part in measurements and 49 players returned at follow-up of whom 47 were included in the analyses (Fig. 1). Players who dropped out were significantly heavier at baseline (mean difference 7.2 kg, 95% CI 0.99 to 13.31, *p* = 0.024), but no other significant differences were seen between drop-outs and those who completed the study. Almost half of the players were active in one or two other sports besides football, most often floorball and handball, and these winter-season sports had minimal overlap with the football season (Table 2). There was a significant difference in weight change from baseline to follow-up between intervention groups, with larger increase in the players in the *Knee Control+* group (1.49 ± 1.17 vs 0.62 ± 1.02 kg, 95% CI 0.22 to 1.52, *p* = 0.010). No adverse events were reported during or after the intervention.

**Table 2:** Demographics for players who took part in both baseline and follow-up measurements

	*Knee Control*	*Knee Control+*
No. of players	21	26
Age years, mean (SD)	13.8 (0.7)	14.5 (0.6)
Years of football experience, mean (SD)	6.1 (2.0)	8.7 (1.8)
Active in other sports, *n*	1 sport (7), 2 sports (2)	1 sport (10), 2 sports (4)
Football profile at school, n	10	12
Other sports profile at school, *n*	2	6
No. of football training sessions/week at baseline, mean (SD)	4.6 (1.1)	4.5 (1.4)
Perceived training volume at baseline, mean (SD)^a^	6.1 (1.0)	5.7 (1.0)
Previous experience of using the *Knee Control* IPEP, *n*	Yes, regularly (0), yes, sporadically (4), no (16), missing (1)	Yes, regularly (0), yes, sporadically (8), no (18)

Values are mean and standard deviation (SD) or n

*IPEP* injury prevention exercise programme

^a^Likert scale 1–7, where 1 represents extremely low training volume and 7 extremely high training volume

The teams reported using the intervention for 15–30 min per session, 2–3 times per week during 5–8 weeks. The team that had the longest IPEP duration per session chose to use the programme on two out of three training sessions per week, and those with shorter IPEP duration used it on all training occasions instead. The teams performed a median of 13 IPEP sessions (range 11–21 sessions). One of the teams in the *Knee Control+* group cancelled training sessions due to heavy rain and waterlogged football grounds during 2 weeks. All teams progressed the training after 2 to 4 weeks of training.

When analysing all complete cases with baseline and follow-up data, no performance improvements were seen from baseline to follow-up when analysing the sample as a whole, and instead, small deteriorations were seen in the agility t test, CMJ and 10-m sprint (Table 3). When analysing the intervention groups separately, small improvements were seen over time in the 505 agility test and side-hop test on the right leg in the *Knee Control* group. The only between-group difference was found in the 505 agility test with a significant interaction effect between the 505 agility test and group allocation, *F*(1,44) = 10.42, where players in the *Knee Control+* group performed worse over time and players in the *Knee Control* group improved their performance over time, Δ_KC vs KC+_ = − 0.012 (95% CI − 0.19 to − 0.04), *d* = 0.98. The results of the sensitivity analysis including all players participating at baseline measurements did not differ substantially from the complete case analysis (data not shown).

**Table 3:** Results at baseline and follow-up and comparisons of change between groups

		Mean (95% CI)			Between-group difference in change (95% CI); *p* value; Cohen´s *d*
		Whole sample	*Knee Control*	*Knee Control+*	
Agility t test (s)	Baseline (1)	11.89 (11.68 to 12.11)	12.24 (11.92 to 12.56)	11.61 (11.33 to 11.89)	
	Follow-up (2)	12.03 (11.82 to 12.24)	12.30 (11.97 to 12.63)	11.81 (11.52 to 12.1)	
	Change (2)-(1)	**0.14 (0.02 to 0.25)**	0.06 (− 0.12 to 0.25)	**0.20 (0.04 to 0.37)**	− 0.139 (− 0.402 to 0.124); *p* = 0.293; *d* = 0.32
Single-leg hop for distance, right leg (cm)	Baseline (1)	143.15 (138.92 to 147.38)	140.4 (133.6 to 147.2)	145.3 (139.2 to 151.3)	
	Follow-up (2)	143.40 (138.35 to 148.46)	139.6 (131.4 to 147.7)	146.4 (139.2 to 153.6)	
	Change (2)-(1)	0.26 (− 2.62 to 3.13)	− 0.87 (− 5.53 to 3.79)	1.16 (− 2.97 to 5.3)	− 2.034 (− 8.637 to 4.569); *p* = 0.538; *d* = 0.191
Single-leg hop for distance, left leg (cm)	Baseline (1)	143.73 (138.49 to 148.98)	139.7 (131.3 to 148.1)	146.9 (139.4 to 154.4)	
	Follow-up (2)	144.43 (138.91 to 149.94)	140 (131.2 to 148.9)	147.9 (140 to 155.7)	
	Change (2)-(1)	0.69 (− 3.19 to 4.57)	0.35 (− 5.98 to 6.67)	0.97 (− 4.64 to 6.58)	− 0.623 (− 9.589 to 8.343); *p* = 0.889; *d* < 0.02
505 agility test (s)	Baseline (1)	2.60 (2.55 to 2.64)	2.63 (2.56 to 2.7)	2.57 (2.5 to 2.63)	
	Follow-up (2)	2.61 (2.57 to 2.65)	2.58 (2.51 to 2.65)	2.63 (2.57 to 2.69)	
	Change (2)-(1)	0.01 (− 0.02 to 0.05)	− **0.05 (**− **0.1 to** − **3.18)**	**0.07 (0.02 to 0.11)**	− *0.118 (*− *0.192 to* − *0.044); p = 0.002; d = 0.975*
Side-hop right (*n*)	Baseline (1)	37.75 (34.33 to 41.16)	37.63 (32.07 to 43.19)	37.83 (32.9 to 42.76)	
	Follow-up (2)	39.28 (35.31 to 43.24)	42.71 (36.38 to 49.03)	36.50 (30.89 to 42.11)	
	Change (2)-(1)	1.53 (− 1.63 to 4.70)	**5.08 (0.11 to 10.05)**	− 1.33 (− 5.74 to 3.07)	6.414 (− 0.633 to 13.46); *p* = 0.073; *d* = 0.553
Side-hop left (*n*)	Baseline (1)	35.61 (31.65 to 39.57)	34.21 (27.8 to 40.62)	36.77 (30.97 to 42.58)	
	Follow-up (2)	37.83 (33.69 to 41.96)	37.00 (30.28 to 43.72)	38.51 (32.43 to 44.59)	
	Change (2)-(1)	2.22 (− 0.71 to 5.15)	2.79 (− 1.97 to 7.54)	1.74 (− 2.56 to 6.04)	1.049 (− 5.792 to 7.89); *p* = 0.759; *d* = 0.09
10-m sprint (s)	Baseline (1)	1.88 (1.85 to 1.91)	1.88 (1.83 to 1.92)	1.87 (1.83 to 1.92)	
	Follow-up (2)	1.90 (1.86 to 1.93)	1.91 (1.85 to 1.96)	1.89 (1.84 to 1.94)	
	Change (2)-(1)	**0.02 (0.01 to 0.04)**	**0.03 (0 to 0.06)**	0.01 (− 0.01 to 0.04)	0.014 (− 0.029 to 0.057); *p* = 0.521; *d* = 0.191
20-m sprint (s)	Baseline (1)	3.37 (3.31 to 3.42)	3.38 (3.28 to 3.47)	3.36 (3.28 to 3.44)	
	Follow-up (2)	3.39 (3.33 to 3.45)	3.39 (3.29 to 3.49)	3.39 (3.31 to 3.48)	
	Change (2)-(1)	0.03 (− 0.00 to 0.05)	0.02 (− 0.03 to 0.06)	0.03 (− 0.01 to 0.07)	− 0.016 (− 0.078 to 0.047); *p* = 0.616; *d* = 0.155
Countermovement jump (m)	Baseline (1)	0.25 (0.24 to 0.26)	0.24 (0.22 to 0.26)	0.25 (0.24 to 0.27)	
	Follow-up (2)	0.24 (0.23 to 0.25)	0.23 (0.21 to 0.25)	0.25 (0.23 to 0.26)	
	Change (2)-(1)	− **0.01 (**− **0.02 to 0.00)**	− 0.01 (− 0.02 to 0)	− 0.01 (− 0.02 to 0)	− 0.002 (− 0.02 to 0.015); *p* = 0.801; *d* = 0.063

Age-adjusted results are reported for the two intervention groups at baseline and follow-up, and their change between time points, as well as between-group differences in change, from the intention to treat analysis. Bold text indicates a statistically significant within-group difference from baseline to follow-up *p* < 0.05. Text in italic indicates a statistically significant between-group difference in change *p* < 0.05.

## Discussion

This study showed no meaningful performance improvements after 5–8 weeks of preventive training exercises and no meaningful differences in effects between interventions. There was a significant difference in change between groups for the 505 agility test with reduced performance in the *Knee Control+* group and an improvement in the *Knee Control* group. Even though the effect size was large, the between-group difference (0.07 s) was too small to be of any clinical importance.

### Performance Effects of IPEP Training

Our findings on youth male football players in the current study are in line with a previous study on *Knee Control* where no effect was seen on performance parameters in youth female football players [17]. However, the lack of positive performance effects from *Knee Control* is in contrast to previous studies in youth male players evaluating the 11+ and 11+ Kids IPEPs [1, 9–14]. Importantly, however, players were younger in four of these studies [1, 9, 11, 14], which may in part explain the differing results since the magnitude of effects of strength and power training has been shown to be larger among children compared to adolescents [25]. Since the 11+/11+ Kids and the *Knee Control* IPEPs contain very similar exercises, and all have been shown to be efficacious in preventing injuries [15, 26, 27], there is no reason to believe that *Knee Control* works through different effect mechanisms than these comparable programmes. It is, however, important to keep in mind that in earlier studies showing performance improvements after IPEP training the effect sizes were usually rather small [1] and studies included few players. Additionally, the specificity between testing and training was rather low in this study, as well as earlier studies, with tests of explosive muscle actions and sprinting but mostly exercises with slow controlled movements, especially in the *Knee Control* group.

The few changes seen within the intervention groups in the present study were mostly negative with small declines in sprint and agility performance over time. Deteriorations were also seen in the *Knee Control+* group despite this programme version being more demanding and with more plyometric elements. It is likely that the declines in performance are related to the timing of the study period, with the intervention implemented at the end of the football season with players rating their training volume as high, already at baseline, and feeling worn out after a long season. This is corroborated by studies showing reduced strength after a short period of match congestion [28, 29]. Half of the players also had a football profile at school, adding to the overall amount of training. Considering this, the results are positive showing that a preventive programme may be added to the overall football training regime without compromising performance. The main aim of the programmes is, after all, to prevent injuries. During these periods of heavy training load, teams may feel tempted to reduce the preventive training to avoid overexertion, whereas this study shows that it is safe to continue with preventive training without compromising performance. The teams had used the intervention on 11–21 sessions during the intervention period, i.e. roughly one training session per week, the same as the training dose shown to reduce the risk of anterior cruciate ligament injury in youth female football players [15]. The study duration was quite similar to the other studies on performance effects [1, 9, 11–14]. As in our earlier studies in youth football [17, 30], low player training attendance was the main reason for limited player IPEP compliance and training dose, which is hard to counter in this study context.

### Methodological Considerations

Methodological strengths of the study were true randomisation, blinded assessors, standardised testing, use of practice trials before testing to minimise influence of learning effects and use of technical devices to record sprint and agility times and jump height. The main limitation is the relatively short follow-up of 5 to 8 weeks. With a longer intervention period and another timing of the intervention, the scope for improvement would probably have been greater since there would have been more time to progress the training. Other limitations were the lack of a true control group without any intervention to assess the influence of e.g. physical development on performance outcomes, the short time for progressing the programme, the rather low intervention training dose and lack of individual data on preventive training. Finally, there is a risk of selection bias in that the most fit or motivated players might have returned for follow-up testing while those less fit or motivated, but probably more likely to improve from the interventions, dropped out.

## Conclusion

In conclusion, no clinically meaningful improvements on sport-relevant performance tests were seen after 5–8 weeks of injury prevention exercise training in male youth football players. Differences in effects on performance tests between *Knee Control* and *Knee Control+* seemed to be minor.

**Table:** 

Principal exercise/focus	*Knee Control*		*Knee Control+*	
	First 2 weeks	Progression	First 2 weeks	Progression
One-legged knee squat	A) With the hands on the hips	B) Straight arms above the head C) With foot marking at 12-02-04-06 o’clock positions D) Diagonal movement with ball E) Partner exercise: Two teammates pressing the ball between them with one foot each while squatting	- Same as the original programme - Partner exercise: single leg standing and pulling/pushing each others’ hands - One-legged knee squats standing in a ring throwing a ball to each other	- One-legged knee squats standing in a ring throwing/kicking a ball to each other - Partner exercise: one-legged squatting while kicking a ball back and forth - Deep one-legged knee squats (as deep as possible)
Hamstring strengthening	A) Pelvic lift with both feet on the ground	B) Single leg pelvic lift with one foot on the ground C) Single leg pelvic lift with one foot on the ball D) Static single leg pelvic lift with explosive change of supporting foot E) Partner exercise: single leg pelvic lift with working leg supported by teammate	- Crab walk - Pelvic lifts (same as the original programme) - Static pelvic lift with changing foot position forwards/backwards	- The diver: standing on one leg flexing and extending the hip while holding the ball and reaching towards the ground and standing straight respectively - Pelvic lifts (same as original programme) - Partner exercise: Prone position single leg-curls with teammate resisting the curl - Partner exercise: Nordic hamstring exercise
Two-legged knee squat	A) Holding a ball with straight arms in front of the body	B) With hands on the hips C) Straight arms above the head and holding a ball D) Same as C but with toe raises when extending the legs E) Partner exercise: holding the ball with one hand each while squatting	- Walking forward in a squatting position - Partner exercise: Two-legged knee squats with ball kicks - Two-legged knee squats (same as the original programme)	- Countermovement jumps - Two-legged knee squats (same as the original programme) - Deep plyometric two-legged jumps forward
Core strength	A) The plank with knee support	B) The plank with foot support C) The plank with foot support and foot marking from side to side D) Dynamic side plank E) Partner exercise: the wheelbarrow	- The plank with foot support - Partner exercise: The plank facing each other and clapping hands - Bear walk - - Partner exercise: Standing core strength holding a ball where one teammate tries to shake the ball and the other tries to hold still	- Partner exercise: standing core strength by leaning towards each other while holding palms against the teammates’ palms - Supine core exercise with bent knees trying to dip one foot at a time towards the ground - Trunk rotation lying on the ground rolling from supine to prone position with the whole body held straight
Lunges	A) Walking lunges with hands on the hips	B) Walking lunges with trunk rotation holding the ball C) Walking lunges holding a ball above the head D) Sideways lunges with straight arms in front of body E) Partner exercise: Lunges at the same spot with throw-ins to teammate	- Lunges upwards downwards while standing still - Walking lunges across the ground with hands on the hips - Partner exercise: Lunges at the same spot with throw-ins or headings to teammate	- Lunges (same as the original programme) - Scissor jumps - Team exercise, team positioned in two lines facing each other: Lunges on the spot with throw-ins or headings to the teammate on the opposite side
Jump/landing	A) Single leg jump and landing forwards and backwards	B) Skating hops side to side C) Fast jogging and stop and hold forwards D) Fast jogging and stop and hold sideways E) Partner exercise: squat jumps while heading the ball (thrown by the teammate)	- Two-legged jumps throwing and catching a ball in different directions - Two-legged jumps in different directions - Team exercise: “follow the leader” with two-legged jumps	- Jump/landing (same as the original programme) - Running higgledy-piggledy and jumping and clapping hands or jumping as for a heading duel when meeting teammate - One-legged hops in a square

## Data Availability

The data sets used during the current study are not publicly available but are available from the corresponding author on reasonable request.

## Appendix

Description of the interventions and their progressions

## Abbreviations

CMJ: Countermovement jump
ICC: Intraclass correlation coefficient
IPEP: Injury prevention exercise programme
